# Volatility and Persistence of Value-Based Purchasing Adjustments: A Challenge to Integrating Population Health and Community Benefit Into Business Operations

**DOI:** 10.3389/fpubh.2020.00165

**Published:** 2020-06-09

**Authors:** Jason S. Turner, Kevin D. Broom, Kenton J. Johnston, Steven W. Howard, Susan L. Freeman, Travis Englund

**Affiliations:** ^1^Department of Health Services Management, Rush University, Chicago, IL, United States; ^2^Department of Health Policy and Management, University of Pittsburgh, Pittsburgh, PA, United States; ^3^Department of Health Management and Policy, Saint Louis University, Saint Louis, MO, United States; ^4^Department of Internal Medicine, Rush University, Chicago, IL, United States; ^5^Bryan Memorial Hospital, Lincoln, NE, United States

**Keywords:** value-based payment, population health, community benefit, healthcare financing, payment methodologies

## Abstract

With the passage of the Deficit Reduction Act of 2005 and the Patient Protection and Affordable Care Act in 2010, Medicare's Inpatient Prospective Payment System (IPPS) began a transition to value-based purchasing (VBP) that rewards or penalizes hospitals based on patient satisfaction, clinical processes of care, outcomes, and efficiency metrics. However, hospital-level volatility vs. persistence in value-based payments year-over-year could result in unpredictable cash flows that negatively influence investment behavior, drive underinvestment in community benefit/population health management initiatives, and make management of the factors that drive the VBP adjustment more challenging. To evaluate the volatility and persistence of hospital VBP adjustments, the sample includes VBP adjustments and the associated domain scores for the 2,547 hospitals that participated in the program from 2013 to 2016. The sample includes urban (74%), teaching (29.1%), system affiliated (46.5%), and not-for-profit (63.6%) facilities. Volatility was measured using basic descriptive statistics, relative risk ratios, and a fixed effect, autoregressive, dynamic panel model that robust-clustered the standard errors. There is substantial change in a given facility's total VBP score with an average standard deviation of 10.74 (on a 100-point scale) that is driven by significant volatility in all metrics but particularly by efficiency and outcomes metrics. Relative risk ratios have dropped substantially over the life of the program, and there is low persistence of VBP scores from one period to the next. Findings indicate that if hospitals receive a positive adjustment in 1 year, they are almost as likely to receive a negative adjustment as a positive adjustment the following year. Furthermore, using a fixed-effect dynamic panel model that controls for autocorrelation, we find that only 13.5% of a facility's prior year IPPS adjustment (positive or negative) carries forward to the next year. The low persistence makes investment in population health management and community benefit more challenging.

## Introduction

National healthcare expenditures have grown from $146 per person in 1960 to $11,172 per person in 2018. During the same time period, the percentage of the gross domestic product (GDP) devoted to healthcare has grown from 5% to over 17.7%. Healthcare spending projections from Centers for Medicare and Medicaid Services (CMS) continue to grow at rates that outstrip projected inflation rates and are projected to account for per person spending just under $17,000 (almost 20% of the GDP) in the next 7 years (1).

Payers, both public and private, have responded to the expense growth by altering incentives, manipulating benefits, increasing cost sharing, and limiting provider networks all in attempts to constrain risks and expense growth rates. More recently, there is a movement to accountable care organizations, shared savings programs, and value-based payments. There is also increasing attention being paid to community benefit reporting and the promise of community benefit and population health management (2, 3).

Not a new concept, population health focuses on “the health of a population as measured by health status indicators and as influenced by social, economic, and physical environments, personal health practices, individual capacity and coping skills, human biology, early childhood development, and health services. As an approach, population health focuses on interrelated conditions and factors that influence the health of populations over the life course, identifies systematic variations in the patterns of occurrence, and applies the resulting knowledge to develop and implement policies and actions to improve the health and well-being of those populations” (4). The interplay between the social determinants of health, the larger environment, and population health is well-documented by Kindig and Stoddart (5), McAlerney (6), the World Health Organization, and a host of more recent research (7). What is not clear is how healthcare payers and systems can successfully initiate and sustain population efforts while enhancing long-term viability and support within the new payment framework.

The Centers for Medicare and Medicaid Services (CMS) is moving more Medicare fee-for-service (FFS) reimbursements to an alternative payment model (APM) basis and aims to grow that percentage to 50% (8, 9). To date, CMS has instituted programs that identify and disseminate best practices, established bundled payments for comprehensive episodes of care, held providers responsible for total cost of care and overall quality, and established pay-for-performance (P4P) rewards and penalties for provider performance relative to preset metrics. With the passage of the Deficit Reduction Act of 2005 and the Patient Protection and Affordable Care Act in 2010, Medicare's Inpatient Prospective Payment System (IPPS) began an APM transition to value-based purchasing (VBP). The VBP legislation and subsequent CMS rules are intended to move hospitals from a payment system in which facilities are financially rewarded for volume to a P4P system that accounts for patient experiences, adherence to predetermined clinical protocols, health outcomes, and cost efficiency in the delivery of care.

Though voluntary in 2012, participation in the VBP program became mandatory in 2013 for hospitals receiving IPPS payments and meeting the minimum number of cases, surveys, or measures required to calculate the adjustment (psychiatric, rehabilitation, long-term care, children's, and cancer hospitals are exempt). The program works by adjusting the Diagnosis-Related Group (DRG) base rate up or down relative to performance on predetermined measures. Adjustments started at ±1% of IPPS hospital-specific base rates in 2013 and have increased by 0.25% per year. In 2017, the program put up to 2% of the Medicare IPPS at risk—the maximum at-risk percentage for the program. Because the program is revenue neutral, increases in the hospital base rate are equally offset by decreases at other hospitals with the average adjustment centered on zero. Those facilities that deviate the most positively or negatively from the mean receive the largest positive and negative IPPS adjustment.

In an environment where profit margins are already thin, ranging from 3 to 5% depending on hospital ownership, location, and teaching status (10, 11), fluctuations in the IPPS can have a direct and immediate impact. Moreover, the impact of the Medicare changes can then be compounded by commercial payers who tend to use Medicare payments and associated adjustments as a baseline for contractual language and payments.

While some prior work has examined the magnitude of the VBP adjustments and the associated relationships with quality and hospital profitability (12–14), this article attempts to quantify the volatility and persistence of VBP adjustments for participating facilities in the early years of the program. Previous research has addressed quality outcomes of the VBP program and the magnitude of the VBP adjustments; however, this study is the first to investigate the volatility and persistence of VBP payments since the inception of the program. Volatility in P4P payment results in unpredictable cash flows that negatively influence investment behavior (15, 16) and make management of the factors that drive the VBP adjustment more challenging (17, 18). In the balance of this article, we provide a brief review of the VBP/P4P literature before presenting: (1) an examination of the volatility of VBP adjustments as well as the components that influence the composite score, (2) a calculated measure of persistence that quantifies how much of a facility's prior year's adjustment carries forward to the next year, and (3) a discussion of the volatility and persistence of payments on community benefit and population health.

## Literature Review

Prior literature on earnings persistence within the healthcare sector is largely absent. Outside of the sector, substantial efforts have been devoted to the relationship between measures of persistence and methods of improving security pricing (19–21), the negative impacts of earnings volatility on investment behavior (15, 16), the higher costs of capital and lower capital investment associated with low earnings persistence (22), and the impact on accounting accruals (23).

The prior research on the effects of P4P programs, including systematic reviews, is more robust, but the findings are mixed (24). Some studies find no difference in health outcomes (25), whereas others have documented improvements in composite measures of quality (26). More recently, the Quality Incentive Program, the Medicare VBP program that is associated with end-stage renal disease, notes substantial improvement in clinical process measures (27). Briesacher et al. (28) also found that P4P increased access and improved outcomes in nursing facilities but increased costs. Several survey studies have shown P4P initiatives to be cost effective; however, the associated interventions have tended to be narrowly focused. Among the more narrowly defined P4P initiatives, Armour and Pitts found that physician bonuses/withholds reduced outpatient expenditures by 5% (29). Existing literature shows that the cost-effectiveness of a program appears to depend on the design of the interventions and incentives (30).

Despite the potential for adjustments of up to 1.75% in 2016, early evaluations of the VBP adjustment indicate that over 74% of hospitals nationally experience a change in IPPS reimbursement of >0.50% (12, 13, 31). Financially, earlier work did not find a significant relationship between VBP adjustments and facility profitability in the early years of the program, and there was no apparent change in quality of care (14, 32). More recently, Ryan et al. (33) found that there was no significant relationship between the aggregate VBP adjustment and improvements in patient experience or quality of care metrics. In some cases, favorable VBP adjustments are related to poor performance on metrics that are costly to improve being offset by savings in expense-related metrics (34). There has also been no relationship between bond rating and the factors that influence the VBP adjustment, with the exception of Medicare spending per beneficiary (MSPB) (35). From a bond perspective, Rangnekar et al. (35) found a positive relationship between high levels of MSPB, which will result in downward VBP adjustments, but favorable bond ratings, which will decrease the borrowing costs for facilities. Ironically, hospitals that operate more efficiently to improve their VBP adjustments will hurt their bond ratings in the process, resulting in a reduced ability to secure funding for furtherance of the organizational mission. Not surprisingly, hospitals affiliated with systems, that are able to learn from others, and that have a level of market control do better than their counterparts in hospital VBP adjustments (36). There also appears to be a significant and negative relationship between particular hospital lines of business and the hospital VBP adjustment. Trauma certified hospitals consistently score poorer on VBP metrics (37).

## Overview of P4P Payment Incentives for Hospitals

Unlike some prior P4P payment incentives that employ more targeted performance metrics and incentives, the VBP adjustment to the IPPS utilizes a wide variety of factors. In 2013, the first year of the program, the VBP adjustment was driven only by patient satisfaction and clinical process scores, with 70% of adjustment driven by the clinical process score. As the program matured, outcomes and efficiency metrics were added to the overall calculation and accounted for 40 and 25% of the overall adjustment, respectively. As detailed in Table 1, the 2013–2016 adjustments fall into four categories: person and community engagement, clinical processes of care measures, safety, and a measure of efficiency that is scored on a 0–100 scale (facilities at the top of the distribution receive a score of 100 and those at the bottom receive a score of 0). The 2016 program split 23 distinct measures across all four domains. The content of each of these VBP categories is highly varied and ranges from patient satisfaction with nurse communication and the cleanliness of the facility to central line–associated bloodstream infections and spending per Medicare beneficiary^1^.

**Table 1:** Value-based purchasing domains, measures, and weighting 2013–2016.

	**VBP components**	**2013**	**2014**	**2015**	**2016**
Patient experience (HCAHPS)	Nurse communication	30%	30%	30%	25%
	Doctor communication				
	Responsiveness of staff				
	Pain management				
	Communication of medicine instructions				
	Hospital cleanliness and quietness				
	Discharge Information				
	Overall rating				
Clinical process of care measures	Fibrinolytic therapy within 30 min of hospital arrival (Acute Myocardial Infarction)	70%	45%	20%	10%
	Primary PCI received within 90 min of hospital arrival (Acute Myocardial Infarction) (Discontinued for 2016)				
	Discharge instructions for patients (Heart Failure) (Discontinued for 2016)				
	Blood cultures performed in ED prior to initial antibiotic (Pneumonia) (Discontinued for 2016)				
	Initial antibiotic selection for CAP in immunocompetent patient (Pneumonia)				
	Prophylactic antibiotic received within 1 hr prior to surgical incision (Healthcare-Associated Infections) (Discontinued for 2016)				
	Prophylactic antibiotic selection for surgical patients (Healthcare-Associated Infections)				
	Prophylactic antibiotics discontinued within 24 hrs after surgery end time (Healthcare-Associated Infections)				
	Cardiac surgery patients w/controlled 6 AM postoperative serum glucose (Healthcare-Associated Infections) (Discontinued for 2016)				
	Post-operative urinary catheter removal on post-operative day 1 or 2 (New in 2014)				
	Surgery patients on a beta blocker prior to arrival who received a beta blocker during the perioperative period (Surgical Care Improvement)				
	Surgery patients who received appropriate venous thromboembolism prophylaxis within 24 hrs prior to surgery to 24 hrs after surgery (Surgical Care Improvement)				
	Surgery patients w/recommended venous thromboembolism prophylaxis ordered (New in 2014 - Discontinued in 2015)				
	Influenza Immunization (New in 2016)				
Outcome measures	Acute myocardial infarction 30-days mortality rate		25%	30%	40%
	Heart failure 30-days mortality rate				
	Pneumonia 30-days mortality rate				
	Composite patient safety indicator (New in 2015)				
	Central Line-Associated Bloodstream Infections (New in 2015)				
	Catheter-Associated Urinary Tract Infection (New 2016)				
	Surgical Site Infection: • Colon • Abdominal Hysterectomy (New 2016)				
Efficiency	Medicare spending per beneficiary			20%	25%
	Potential Medicare IPPS adjustment to base rate	1.00%	1.25%	1.50%	1.75%

## Methods

### Data

VBP adjustments and the respective unweighted domain scores for all hospitals in the United States were gathered from CMS for years 2013–2016. For descriptive and analytic purposes, hospital characteristics were gathered from HCRIS data (Hospital Form 2552-10) and matched on the unique provider identification. Inclusion criteria required VBP adjustments for all 4 years of the program and resulted in 2,547 hospitals and 7,641 observations. Hospital characteristics of those in the analysis are included in Table 2 and include 742 teaching facilities, 1,184 facilities with system affiliations, 1,620 not-for-profit facilities, and 1,886 urban facilities. The analysis is limited to the 2013–2016 time frame due to reporting changes instituted by CMS. In more recent years, CMS substantively changed how domain measures were shared such that weighted and unweighted composite scores by domain were discontinued and replaced with a metric-specific number between 1 and 10. One of this study's limitations is that the newer scores do not carry the same resolution as the prior scale (0–100), are not aggregated at the domain level, and, as a result, limit the study's framework to the earlier years of the program.

**Table 2:** Hospital sample composition (*n* = 2,547).

Urban	1,886	74.0%
Teaching	742	29.1%
System Affiliated	1,184	46.5%
Not-For-Profit	1,620	63.6%

### Measures

The volatility of the IPPS adjustments was measured as the standard deviation and the relative standard deviation (standard deviation/mean) of their final VBP score (scored 0–100) prior to a financial adjustment being tied to a given score. The process was repeated for the associated VBP domains over the 2013–2016 time frame as long as the domain contributed to the final IPPS adjustment. Since unweighted domain scores are scored based on a 0–100 percentile achievement for all years in the study, they did not require standardization. However, the financial VBP adjustments increased from ±1 to ±1.75% over the 2013–2016 time frame and required standardization. To account for the change, every facility adjustment was standardized by the total potential adjustment in the respective year for a standardized adjustment of between−100 and 100% for every year in the sample. For example, a facility that received an upward adjustment of 0.75% in 2014 would receive a standardized score of 60% or (0.75/1.25). With an upward adjustment of 0.75, the facility received a total of 60% of the total potential upward adjustment for that time period.

Relative risk measures were also calculated for the 2013–2014, the 2014–2015, and the 2015–2016 time frames as an additional volatility metric. These metrics measure the relative risk of receiving a positive VBP adjustment where receiving a positive adjustment in the prior year is treated as the exposure. All measures of volatility are presented in Table 3.

(1) $$\begin{array}{r} {Standardized\;Adjustment}_{it}=Intercept\\ \;+\beta_{1}{Standardized\;Adjustment}_{i\left(t-1\right)}\\ \;+\;\beta_{n}Vector\;of\;time\;invariant\;\\ \;hospital\;characteristics\;+\;error \end{array}$$

Persistence of the VBP adjustment was measured as the β_1_ coefficient associated with a lagged, standardized VBP adjustment in a time series analysis (a dynamic panel model with maximum likelihood estimation) where time-invariant hospital characteristics are fixed (Equation 1). The standardized adjustment for a given period *t* and facility *i* serves as the dependent variable. The standardized score from the hospital's prior period (*t-1*) and a vector of time-invariant, hospital-specific characteristics serve as the independent variables. Standard errors were clustered at the facility level to adjust for within-facility correlations after Durbin–Watson tests indicated some small autocorrelations (38). The fixed effect and between group analysis of persistence is presented in Table 4. The analysis was also repeated with the unique provider identification serving as the fixed effect and found no differences in the estimates or standard errors.

**Table 3:** Relative risk and average standard deviation of hospital total and domain scores.

	**Standard deviation**	**Coefficient of variation**
Overall score	10.74	
Patient experience	8.56	0.247
Clinical processes of care	12.19	0.23
Outcomes	16.11	0.422
Efficiency	22.12^*^	1.19^*^
Relative risk ratio of receiving a positive adjustment given a positive adjustment in the prior year			
2013–2014	3.159	
2014–2015	1.499	
2015–2016	1.012	

* *Excludes facilities where no efficiency score is calculated by CMS in both 2015 and 2016*.

**Table 4:** Dynamic panel regression with fixed effects.

	**Within group estimates w/fixed effects**				**Between group estimates w/fixed effects**			
	**Parameter estimate**	**Standard error**	**T-value**	***P*-value**	**Parameter estimate**	**Standard error**	***T*-value**	***P*-value**
Intercept	0.02558	0.000063	405.4	<0.0001	0.076282	0.00541	14.11	<0.0001
Prior year score	0.13527	0.015989	8.46	<0.0001	0.890304	0.0112	79.82	<0.0001
System affiliation					−0.01298	0.00456	−2.85	0.0045
Not-for-profit					0.016326	0.00455	3.59	0.0003
Teaching					−0.02617	0.00512	−5.11	<0.0001
Urban					−0.06468	0.00527	−12.26	<0.0001
		R-squared		0.6256		R-Squared		0.7338

## Results

The VBP adjustments are, by design, closely centered on zero with an average of 51.3% of facilities receiving a positive adjustment between 2013 and 2016. Over the 4 years of the study, facilities have experienced substantial variation in their total VBP score. The average facility has a standard deviation of 10.74 and an associated standardized standard deviation of 0.2438. Although each domain has variation that is not inconsequential, the deviations in total score appear to be driven by volatility in the efficiency and outcomes domains, which have standard deviations of 22.12 and 16.11, respectively.

Relative risk calculations also indicate substantial variation in VBP scores and the associated payment adjustments. If a facility received a positive adjustment in 2013, they were 3.159 times more likely to receive a positive adjustment in 2014. That positive association greatly attenuated over the subsequent years. Between 2014 and 2015, the same calculation yielded a relative risk of 1.499. By 2015–2016, a relative risk of 1.0118 indicates that facilities were almost as likely to receive an IPPS penalty despite receiving a positive adjustment in the prior year.

The persistence measure associated with VBP adjustments reinforces the volatility metrics. In the fixed-effects model where hospital characteristics and autocorrelation are controlled for, a β_1_ of 1 would indicate that the facility received the same standardized adjustment in the current year that they received in the prior year. In short, persistence would be high since hospitals would receive the same standardized VBP adjustment from one period to the next. The prior year adjustment is a significant predictor of current adjustments (*P* < 0.0001), and our model estimated a β_1_ parameter of 0.135. On average, 13.5% of a hospital's prior VBP financial penalty or reward carries to the next period.

When examined on a between-group basis, all of the time-invariant characteristics are significant predictors of the VBP adjustment persistence. Facilities that are affiliated with a system, designated as urban, and are teaching institutions have, on average, maintain slightly less of their VBP adjustments than peers. Not-for-profit firms maintain slightly more of their VBP adjustment. While all characteristics are significant at the <0.01 level, the adjustments are not operationally significant. The largest between-group difference is among urban and non-urban facilities where the parameter estimate is −0.0646. To put this in context, a −0.0646 parameter estimate indicates that urban facilities are able to maintain one-tenth of one percentage point (−0.0646 × potential adjustment of 0.0175 in 2016 = 0.0011) more of their VBP adjustment relative to non-urban facilities.

## Discussion

Hospitals participating in the VBP program have experienced significant volatility in their total VBP score and a lack of persistence in the associated IPPS adjustments. Hospitals that receive a positive adjustment in 1 year are now almost as likely to receive a penalty in the next. The lack of consistency from one period to another makes both financial planning and process management more challenging.

As discussed in earlier works (14, 39), because the VBP adjustment is designed to be revenue neutral with adjustments centered on zero, it makes it more difficult for hospitals to differentiate themselves from others participating in the system. To maximize their IPPS payment, facilities must achieve significantly better outcomes, patient satisfaction, and adherence to clinical processes at a lower cost per beneficiary. While above-average achievement in multiple domains is possible, regression to the mean and/or above average performance in one domain offset by below-average performance in another results in relatively tight clustering around zero (32) with over 74% of facilities receiving a bonus or penalty of <0.05%.

The costs of compliance and metric improvement are also important to note. CMS expanded the VBP program to include 23 separate metrics and added a new safety domain (weighted at 20% of overall score) in 2017. As metrics and domains are added, there are at least two direct impacts on hospitals. First, the relative weight of any given metric and the impact it can have on the overall IPPS adjustment diminish. The financial weight and resulting adjustments are spread over more domains and metrics. Second, as items are added to the evaluation protocol, hospitals must implement methods of tracking and improving those metrics^2^. Given the volatility of the scores and adjustment, the investment to report and improve may not generate a return or result in improved quality.

It is not clear that each domain and metric reinforce each other. For example, to score well on the MSPB metric, a facility would be interested in cost control and utilization management tools. As suggested by Das et al. (34), those cost control or utilization management tools may make clinical staff less available for patient interaction and drive down satisfaction scores. It is also conceivable that cost considerations could influence other domains.

From a community benefit perspective, the “two-canoe” problem of population health initiatives becomes more pronounced under the hospital VBP methodology. Providers have one foot in a canoe that is operating in the traditional volume-based system that incentivizes providing more frequent and more expensive care while the other foot is attempting to occupy a canoe operating in a value-based environment where there are efforts to constrain costs and reduce care that has little to no marginal benefit (40). As long as payment systems deploy diverging incentives with low persistence and high volatility, then health systems and providers will have a difficult time investing in community benefit and population health management. The findings this study coupled to the lack of a significant relationship between improvements and VBP adjustments (33) seem to support the diverging incentives put forth by others.

There is also a “wrong pocket problem” with a mismatch between investors and those who accrue the benefits of improved health. Providers and facilities may make significant investments of time and money to improve the health and well-being of the communities they serve and those benefits do not necessarily accrue to the investing providers. Efforts may be effective and even result in fewer patient visits or reduced facility occupancy, which has a negative impact on the bottom line. Not only is the healthcare provider incurring community benefit expenses, but they are also negatively impacted by a reduction of volume and frequency. It is also possible that the benefits of population health initiatives accrue to competing or nearby providers that did not make the investment.

In cases of a shared-savings program, the reduction in volume may be offset by payouts from the program. The programs themselves transfer significant health status (probability of falling ill and needing care) and medical care (cost of providing care) risk without an associated risk premium. Of the 32 pioneer ACOs that pursued shared savings program with CMS, only 8 remain with only 6 receiving a positive payout at an average rate of 1.37% of benchmark expenditures (41). There appears to be a mismatch between the upside of these programs and the risk being borne.

## Conclusion

Although there was great interest in the initial years of the VBP program, each year there exists little financial reward for provider organizations (60% hospitals won or lost <0.5%) (42), and what little opportunity that exists comes at significant cost. Even for providers who earn them, VBP payments are not consistent, leaving organizations unable to plan on receiving them over time, thereby hampering strategic planning and future investment decisions. Moving forward, additional research on the relationships between hospital characteristics and quality metrics should be investigated. Specific and additional attention should be paid to the impact of cost control metrics (MSPB) and patient satisfaction, adherence to clinical guidelines, and outcomes. As the program continues to mature and administrations reevaluate current healthcare legislation, thought should also be given to: (1) increasing the financial incentive to influence behavior, (2) moving to a forced distribution of the VBP adjustment such that more facility revenue is at risk for poor performance, and/or (3) narrowing the number of metrics included in the program to focus facility efforts. An alternative method of improving safety and quality may include a more targeted approach that sets facility-specific performance targets (43).

Payment adjustments continue to be tightly distributed around zero with the majority of hospitals receiving an adjustment of < 12 of percentage. What this means is that facilities may make investments in population health or value-based care but not realize any downstream payments from CMS. The resulting volatility of cash flows discourages investments to improve population, community health, and value metrics.

## Data Availability Statement

The datasets generated for this study may be made available upon request to the corresponding author.

## Author Contributions

All authors listed have made a substantial, direct and intellectual contribution to the work, and approved it for publication.

## Conflict of Interest

The authors declare that the research was conducted in the absence of any commercial or financial relationships that could be construed as a potential conflict of interest.

## Footnotes

^1^The hospital VBP program has continued to evolve and change. The 2020 domains largely remain the same with slight nomenclature changes. The number of metrics within those domains has been reduced to 20 and includes: catheter-associated urinary tract infection, central line-associated blood stream infection, *C. difficile*, methicillin-resistant *Staphylococcus aureus* Bacteremia, elective delivery prior to 39 completed weeks gestation, surgical site infection for colons and abdominal hysterectomies, AMI, heart failure 30-days mortality, pneumonia 30-days mortality, total hip arthroplasty and/or total knee arthroplasty, Medicare spending per beneficiary, communication with nurses, communication with doctors, responsiveness of hospital staff, communication about medicines, hospital cleanliness and quietness, discharge information, three-item care transition, and overall rating of the hospital.

^2^It is important to note that not only have some metrics within domains changed but the weighting of each domain has continued to evolve. In 2020, the impact of each of the four domains on the hospital VBP adjustment is equally weighted.
